# Association of tenacious goal pursuit and flexible goal adjustment with out-of-home mobility among community-dwelling older people

**DOI:** 10.1007/s40520-018-1074-y

**Published:** 2018-11-17

**Authors:** Sini Siltanen, Taina Rantanen, Erja Portegijs, Anu Tourunen, Taina Poranen-Clark, Johanna Eronen, Milla Saajanaho

**Affiliations:** Gerontology Research Center and Faculty of Sport and Health Sciences, University of Jyväskylä, PO Box 35, Viveca 271, 40014 Jyvaskyla, Finland

**Keywords:** Aging, Coping, Mobility, Participation, Autonomy

## Abstract

**Background:**

As people age, functional losses may limit the potential to get outside the home and participate in desired activities and community life. Coping with age-related losses has been reported to be important for psychological well-being. Hitherto is not known whether active use of coping strategies also helps maintain out-of-home mobility.

**Aims:**

We investigated how two coping strategies, tenacious goal pursuit (TGP; persistency in reaching one’s goals) and flexible goal adjustment (FGA; adjusting one’s goals to changed circumstances), are associated with life-space mobility and perceived autonomy in participation outdoors among community-dwelling older people.

**Methods:**

Participants (*n* = 186) were aged 79–93 years. TGP and FGA were self-reported using separate scales. Perceived autonomy in participation was assessed with the Impact on Participation and Autonomy Outdoors-subscale, and life-space mobility with the Life-Space Assessment. Two-step cluster analysis was used to create data-driven coping profiles of TGP and FGA.

**Results:**

General linear model analyses showed that the profile including highly tenacious and flexible older people had the highest life-space mobility and perceived autonomy outdoors, whereas the profile including people with low TGP and low FGA showed the lowest scores. Depressive symptoms attenuated the associations.

**Conclusions:**

Active use of both TGP and FGA is favorable for out-of-home mobility and enables more active participation in society in later life.

**Electronic supplementary material:**

The online version of this article (10.1007/s40520-018-1074-y) contains supplementary material, which is available to authorized users.

## Introduction

Out-of-home mobility is a key element in living an active life in old age. Among community-dwelling older people, leaving the home is associated with greater physical activity [1] and better health and function [2, 3]. Furthermore, going outside the home enables older people to participate in valued activities and community life [4, 5]. Out-of-home mobility can be assessed with life-space mobility, which describes the size of the spatial area a person moves through in daily life, including the frequency of travel and assistance needed for that travel [6]. Thus, life-space mobility describes actual mobility behavior. Out-of-home mobility can also be assessed from a more personal point of view; perceived autonomy in participation outdoors describes an individual’s self-rated possibilities to participate in activities outside the home and takes into account the meaning the individual attaches to these activities [7]. Life-space mobility and perceived autonomy in participation outdoors have been shown to be closely related, but not overlapping, concepts among older people [8].

The ecological theory of aging posits that an individual’s behavior depends on personal competence (e.g. physical and cognitive performance) and environmental press (e.g. obstacles in the living environment) [9]. As physical and cognitive performance typically decline with age, people become more vulnerable to environmental press. This, in turn, may result in imbalanced person-environment fit [9]. In other words, moving around in one’s surroundings becomes more and more difficult, leading to a decline in life-space mobility [10, 11] and perceived autonomy in participation outdoors [12]. However, some people with physical limitations remain highly active, indicating that they may possess other personal resources that enable them to engage in a wide range of activities [13].

Personal goals, i.e. self-selected objectives people want to achieve or avoid [14], guide human behavior [15, 16]. Active striving towards personal goals helps maintain meaningful activities throughout the life span [16] and retain life-space mobility at a higher level with aging [17]. However, age-related functional losses may limit the potential for action, resulting in a need to review and modify one’s goals [16, 18, 19]. Successful goal modification may in turn increase possibilities for participation in new activities. The dual-process model of assimilative and accommodative coping posits two ways of coping with adversity: tenacious goal pursuit (TGP) and flexible goal adjustment (FGA) [18, 20]. TGP, or assimilative coping, refers to persistence and increased effort in adjusting the current situation in line with personal goals, whereas FGA, or accommodative coping, refers to adjusting one’s preferences in response to changes in one’s life circumstances by disengaging from blocked goals or by downgrading their importance [18, 20]. These coping strategies are important for maintaining well-being in old age. Older people who are high in both tenacity and flexibility are less likely to suffer from depression [20–22], a correlate of restricted life-space mobility [23]. Further, older people who actively use both strategies seem to report higher life-satisfaction, and better self-rated health [21, 22].

Potentially, high tenacity and high flexibility may also underlie more active out-of-home mobility among older people. These coping strategies, however, have mainly been studied in relation to psychological outcomes and thus, to our knowledge, their potential role as resources for maintaining out-of-home mobility in later life remains unknown. Hence, we investigated how TGP and FGA are associated with life-space mobility and perceived autonomy in participation outdoors among community-dwelling older people.

## Methods

### Study design

This study used cross-sectional data gathered for the Mobility and Active Aging study (MIIA) conducted at the University of Jyväskylä, Finland. The present participants were randomly selected from among the 848 participants of the Life-Space Mobility in Old Age (LISPE), which was a larger population-based study with a probability sample from the national population register [24]. The present sample was planned to comprise only part of the original LISPE sample, since the power calculations showed that a sample of 200 persons would be sufficient for statistically significant, moderate correlations. Thus, 298 persons were invited to participate. Of these, 77 declined to take part and 15 were not reached. Thus, the present data were gathered from 206 community-dwelling older adults who were aged 79–93 years, able to communicate and living independently in the Central Finland municipalities of Jyväskylä and Muurame.

Of the 848 LISPE participants, the present 206, who also participated in the MIIA study, were somewhat younger (80.0, standard deviation SD 4.1, vs. 80.8, SD 4.3, *p* = 0.02), and had slightly better cognition (Mini Mental State Examination, 26.6, SD 2.3, vs. 26.0, SD 2.9, *p* = 0.01) and physical performance (Short Physical Performance Battery, 10.2, SD 1.8, vs. 9.5, SD 2.7, *p* < 0.001) than the others (*n* = 642). The LISPE + MIIA and LISPE-only participants did not differ by sex, number of chronic conditions, or years of education.

The present data were collected by computer-assisted face-to-face home interviews in spring 2016. In total, 186 participants answered the questions on TGP, FGA, perceived autonomy and life-space mobility. The study protocol was approved by The Ethical Committee of the University of Jyväskylä. Participants signed informed consents before the assessments.

### Measures

#### Tenacious goal pursuit and flexible goal adjustment

Coping was assessed with short versions of the Tenacious Goal Pursuit (TGP) and Flexible Goal Adjustment (FGA) scales, originally developed by Brandtstädter and Renner [20]. The short versions of the scales each contain five items, such as ‘Even when things seem hopeless, I keep on fighting to reach my goal’ (TGP) and ‘If I do not get something I want, I take it with patience’ (FGA) [22]. The response options are consistent with a five-point Likert scale: ‘strongly agree’ (0), somewhat agree (1), doesn’t agree or disagree (2), somewhat disagree (3), and ‘strongly disagree’ (4). There is one inversely phrased item in both scales. Henselmans et al. [25] reported weak face validity for the inversely phrased items. In the present study, the Cronbach’s alphas were higher when the inversely phrased items were omitted (TGP: *α* = 0.77 without vs. *α* = 0.72 with the inversely phrased item, FGA: *α* = 0.67 vs. *α* = 0.60). Moreover, the correlations between the directly and inversely phrased scores were rather low (TGP: *r* = 0.02–0.18, FGA: *r* = 0.09–0.21). Thus, we omitted the inversely phrased items. The remaining four items in both scales were reverse-scored with higher scores indicating higher tenacity or flexibility, and a sum score (range 0–16) was calculated for each scale when responses were given to at least three of the four items. Single missing items were imputed with the mean of the existing values of the respective participant (*n* = 3 in TGP, *n* = 3 in FGA). We excluded 20 participants from the analyses, since they had not responded to any of the questions concerning coping. These participants had lower cognition than the participants included in the analysis (MMSE mean 23.9 vs. 26.1, respectively) and had missing data also in the depression questionnaire (CES-D). We could not use any other time points to estimate responses for these participants.

#### Perceived autonomy in participation outdoors

The ‘autonomy outdoors’ subscale of The Impact on Participation and Autonomy (IPA) questionnaire was used to assess perceived autonomy in out-of-home activities. The IPA is a validated measure, which can be used as a whole or in part (subscales) to assess participation and autonomy [26, 27]. The ‘Autonomy outdoors’ subscale comprises five items on perceived possibilities to (1) visit relatives and friends, (2) make trips and travel, (3) spend leisure time, (4) meet other people, and (5) live life as one wants. The response options range from ‘very good’ (0) to ‘very poor’ (4). A sum score (range 0–20) was calculated with higher scores indicating poorer autonomy.

#### Life-space mobility

Life-space mobility refers to the spatial area an individual purposely moves through in daily life. It factors in all movement irrespective of the mode of transportation and reflects person’s access to community amenities. It was measured with the Finnish version [24] of the University of Alabama at Birmingham Study of Aging Life-Space Assessment (LSA) [6]. The assessment includes six life-space areas starting from the informant’s bedroom and expanding to include the home, yard, neighborhood, town, and beyond town. Participants are asked how often they have moved in each area during the 4 weeks preceding the assessment and whether in doing so, they have needed help from any devices or another person. In the analyses, we used a life-space mobility composite score, which reflects the distance, frequency, and level of independence of mobility, with higher scores (range 0–120) indicating higher life-space mobility [6]. The reliability and validity of the LSA measurement have been established [6, 10].

#### Covariates

In addition to age and sex, which were drawn from the national population register, objectively measured physical and cognitive performance and entrance-related environmental barriers were regarded as theory based confounders. Cognitive performance was assessed with the Mini Mental State Examination (MMSE) [28] and lower extremity function with the Short Physical Performance Battery (SPPB) [29]. Environmental barriers at entrances and in close exterior surroundings were objectively recorded using the Housing Enabler screening tool [30, 31]. Depressive symptoms were assessed with the Centre for Epidemiologic Studies Depression Scale (CES-D) [32].

#### Descriptives

Morbidity was evaluated as the number of self-reported physician-diagnosed chronic conditions from a list of 22 diseases including, e.g. coronary artery disease, diabetes, cancer, and Alzheimer’s disease. An additional open question was asked about conditions other than those on the list [24]. Years of education was also self-reported.

### Statistical analyses

First, the correlations between TGP, FGA, life-space mobility, and perceived autonomy in participation outdoors were tested with Pearson’s correlation. Thereafter, to identify the different coping profiles in our sample, we performed a cluster analysis of TGP and FGA using two-step clustering. Two-step clustering identifies groupings by first running pre-clustering and then running hierarchical methods. Log-likelihood was used as a distance measure and the number of clusters was not determined beforehand. Since cluster solutions can depend on the order of cases, the order was randomized before the analysis. To test the stability of the given solution, cluster analysis was executed four additional times using different randomizations of cases. Differences in background characteristics between the resulting coping profiles were analyzed with chi square test and one-way analysis of variance.

Finally, general linear modeling was used to study the associations of the coping profiles with life-space mobility and perceived autonomy in participation outdoors. The base model was adjusted for age and sex. SPPB, MMSE, environmental barriers, and depressive symptoms were added to the base model one at a time to see which one of them possibly affects the associations. All analyses were performed with SPSS Statistics 24 for Windows.

## Results

Participants’ mean age was 84.0 (standard deviation, SD 4.1) and 56.3% (*n* = 116) of them were women. The mean TGP score across all participants was 11.7 (SD 3.2) and the mean FGA score was 12.3 (SD 2.7). TGP correlated with both life-space mobility (*r* = 0.26) and perceived autonomy in participation outdoors (*r* = − 0.26), whereas FGA correlated only with perceived autonomy (*r* = − 0.27).

**Fig. 1:**
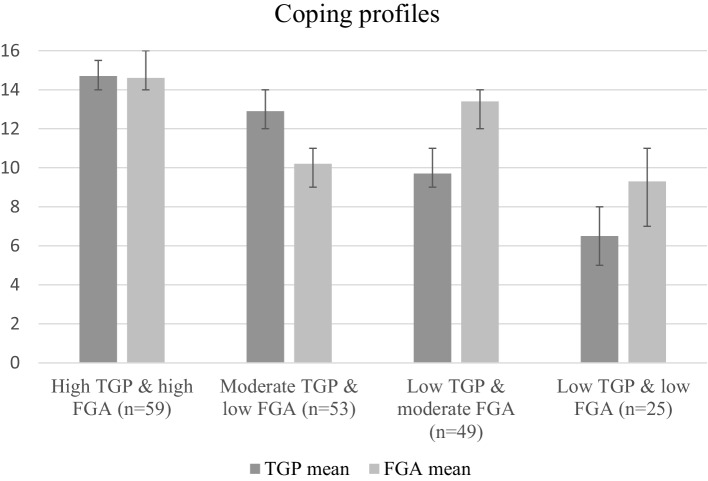
Data-driven coping profiles created with two-step cluster analysis and described with means of tenacious goal pursuit (TGP) and flexible goal adjustment (FGA) in each cluster (ranges 0–16). Error bars represent the interquartile range of the TGP and FGA scores in each cluster

The two-step cluster analysis yielded four coping profiles: (1) high TGP and high FGA (31.7%), (2) moderate TGP and low FGA (28.5%), (3) low TGP and moderate FGA (26.3%), and (4) low TGP and low FGA (13.4%, Fig. 1). The solution remained in four of the five cluster analyses in each of which the participants were differently randomized. The one deviant analysis yielded a three-cluster solution, failing to identify the profile of low TGP and low FGA. Comparison of the participants in the different coping profiles revealed that those with low TGP and low FGA had the poorest scores in cognitive performance, CES-D, life-space mobility, and perceived autonomy, while those high in TGP and FGA reported the least depressive symptoms and restrictions in perceived autonomy, and the highest life-space mobility (Table 1).

**Table 1:** Background characteristics of the participants by coping profile

	Coping profiles				
	High TGP and high FGA *n* = 59	Moderate TGP and low FGA *n* = 53	Low TGP and moderate FGA *n* = 49	Low TGP and low FGA *n* = 25	*p* value
	Mean (SD)	Mean (SD)	Mean (SD)	Mean (SD)	
Age	83.6 (4.0)	83.4 (4.0)	84.1 (4.0)	85.0 (4.3)	0.40^a^
Number of chronic conditions	4.2 (2.6)	4.5 (2.8)	4.8 (2.6)	4.3 (2.0)	0.65^a^
Years of education	9.5 (3.8)	10.3 (5.2)	9.8 (3.5)	10.9 (5.4)	0.57^a^
MMSE	26.6 (2.3)	26.6 (2.2)	26.6 (2.8)	24.9 (3.9)	0.04^a^
SPPB	9.4 (2.0)	9.2 (2.3)	8.9 (3.0)	8.5 (1.9)	0.37^a^
Number of environmental barriers	11.7 (4.0)	11.0 (3.9)	10.8 (4.0)	11.3 (3.2)	0.63^a^
CES-D	6.0 (5.3)	10.7 (7.3)	10.7 (7.0)	12.6 (7.8)	< 0.001^a^
LSA	67.6 (18.2)	61.1 (22.1)	57.2 (21.3)	53.7 (21.4)	0.02^a^
IPA outdoors	4.4 (3.5)	7.2 (3.9)	7.5 (4.2)	8.3 (3.8)	< 0.001^a^
Sex (female) %	52.5	52.8	57.1	64.0	0.77^b^

*MMSE* Mini Mental State Examination, *SPPB* Short Physical Performance Battery, *CES-D* Centre for Epidemiologic Studies Depression Scale, *LSA* Life-Space Mobility Composite Score, *IPA* Impact on Participation and Autonomy Outdoors Score

^a^Analysis of variance

^b^Chi-square test

The general linear models showed that those with low TGP and low FGA had the lowest life-space mobility, except when the model was adjusted for physical or cognitive performance (Table 2). The lowest life-space mobility was then observed among those with low TGP and moderate FGA, while the association with low TGP and low FGA became non-significant. In terms of perceived autonomy in participation outdoors, those with low TGP and low FGA showed the poorest scores even after adjusting for cognitive or physical performance or entrance-related barriers (Table 3). When depressive symptoms were added to the base model, the associations were attenuated in terms both of life-space mobility and perceived autonomy in participation outdoors (Tables 2, 3). For life-space mobility, all the associations became non-significant.

**Table 2:** Marginal means (MM) and standard errors (SE) of life-space mobility scores and regression coefficients (*B*) with 95% confidence intervals (CI) by coping profile

General linear models	Coping profile							
	High TGP and high FGA		Moderate TGP and low FGA		Low TGP and moderate FGA		Low TGP and low FGA	
	MM (SE)		*B*	95% CI	*B*	95% CI	*B*	95% CI
Unadjusted	67.6 (2.7)	Ref.	− 6.57	− 14.27, 1.13	− **10.38**	− 18.25, − 2.51	− **13.91**	− 23.62, − 4.19
Age and sex	66.9 (2.4)	Ref.	− **6.95**	− 13.81, − 0.10	− **9.04**	− 16.05, − 2.04	− **10.35**	− 19.05, − 1.66
Age, sex, SPPB	65.7 (1.9)	Ref.	− 4.52	− 10.13, 1.10	− **6.47**	− 12.16, − 0.77	− 5.77	− 12.91, 1.37
Age, sex, entrance barriers	67.6 (2.5)	Ref.	− **7.98**	− 14.99, − 0.96	− **10.20**	− 17.42, − 2.99	− **10.68**	− 19.58, − 1.77
Age, sex, MMSE	66.5 (2.3)	Ref.	− **6.86**	− 13.45, − 0.28	− **9.19**	− 15.93, − 2.45	− 7.43	− 15.92, 1.06
Age, sex, CES-D	64.9 (2.3)	Ref.	− 3.84	− 10.85, 3.18	− 6.41	− 13.51, 0.70	− 6.69	− 15.58, 2.20

Statistically significant values are bolded

*TGP* tenacious goal pursuit, *FGA* flexible goal adjustment, *SPPB* Short Physical Performance Battery, *MMSE* Mini Mental State Examination, *CES-D* Centre for Epidemiologic Studies Depression Scale

**Table 3:** Marginal means (MM) and standard errors (SE) of perceived autonomy in participation outdoors scores and regression coefficients (*B*) with 95% confidence intervals (CI) by coping profile

General linear models	Coping profile							
	High TGP and high FGA		Moderate TGP and low FGA		Low TGP and moderate FGA		Low TGP and low FGA	
	MM (SE)		*B*	95% CI	*B*	95% CI	*B*	95% CI
Unadjusted	4.4 (0.5)	Ref.	**2.82**	1.39, 4.26	**3.11**	1.64, 4.59	**3.87**	2.06, 5.68
Age and sex	4.5 (0.5)	Ref.	**2.87**	1.49, 4.29	**2.96**	1.54, 4.39	**3.50**	1.74, 5.26
Age, sex, SPPB	4.7 (0.4)	Ref.	**2.58**	1.34, 3.83	**2.59**	1.32, 3.87	**2.86**	1.27, 4.44
Age, sex, entrance barriers	4.5 (0.5)	Ref.	**2.90**	1.48, 4.32	**2.96**	1.49, 4.43	**3.38**	1.57, 5.18
Age, sex, MMSE	4.5 (0.5)	Ref.	**2.87**	1.48, 4.23	**2.98**	1.55, 4.04	**3.31**	1.52, 5.09
Age, sex, CES-D	5.4 (0.4)	Ref.	**1.50**	0.23, 2.76	**1.74**	0.45, 3.02	**1.77**	0.16, 3.37

Statistically significant values are bolded

*TGP* tenacious goal pursuit, *FGA* flexible goal adjustment, *SPPB* Short Physical Performance Battery, *MMSE* Mini Mental State Examination, *CES-D* Centre for Epidemiologic Studies Depression Scale

## Discussion

The findings of this study indicate that older people who persistently pursue their goals, but at the same time are also able to change their goals to better correspond to their current resources, perceive better possibilities to participate in activities outside the home and move across a wider life-space. In contrast, those showing the lowest tenacity and flexibility in pursuing their goals reported the most constraints on out-of-home mobility. This is likely explained by their high prevalence of depressive symptoms. Hence, our findings are not only in line with previous suggestions that being highly tenacious and flexible is the most favorable combination for well-being [21, 22], but extend them to out-of-home mobility. Furthermore, our findings indicate that tenacity is more important than flexibility for life-space mobility. Thus, although the importance of flexibility is often emphasized in later life [18, 33], it seems that tenacity is more crucial when it comes to physically moving around outside of the home.

Both coping strategies aim at reducing discrepancies. According to the dual process model of assimilative and accommodative coping, tenacious persons stay committed to a goal, even when facing hardship, by actively trying to modify the situation to better correspond to their personal preferences and by coming up with new ways of doing things [18]. However, some goals remain unachievable and persistent efforts to strive for them become ineffective. Therefore, flexible goal adjustment is needed to rescale goals within a feasible range or to channel efforts towards new, more feasible goals [18]. Based on the findings of this study, it seems that being persistent but at the same time able to adapt to constraints when necessary, enables people to move across a wider life-space as well as perceive possibilities for doing so. Thus, our findings support the view that people who actively utilize both coping strategies can enjoy the benefits of persistent goal pursuit but also are able to avoid the detrimental effects of persevering in blocked goals [21, 22]. In addition, these results are in line with a previous finding that coming up with new ways of doing things can alleviate environmental press [34].

In contrast, those with the lowest tenacity and flexibility had the lowest life-space mobility and poorest perceived autonomy in participation outdoors. In this profile, restricted life-space mobility was explained by poorer cognitive and physical function. This finding is in accordance with the ecological theory of aging, which posits that moving in one’s surroundings becomes harder as personal competence declines [9]. It is also plausible that functional decline makes it harder to come up with new solutions to overcome obstacles. Furthermore, the association of poorer coping skills with lower life-space mobility was explained by the higher prevalence of depressive symptoms among those who were the least tenacious and flexible. Depression typically causes people to stay inside the home and withdraw from activities [23, 35]. On the other hand, poor coping skills are a risk factor for depressive symptoms, as the individual fails to overcome hardship [20]. Our findings suggest that depressive symptoms may be an essential component of the mechanism between poorer coping skills and restricted out-of-home mobility. However, future studies should examine whether depressive symptoms mediate this association or whether they directly affect coping and out-of-home mobility.

Hardly any previous knowledge exists on the association of TGP and FGA with mobility. However, it has been found that older people who strive for personal goals tend to move across a wider life-space [17], as was also demonstrated in the present study. Previously, TGP and FGA have mainly been studied in relation to psychological outcomes, such as life satisfaction and depression, and it has been suggested that flexibility is the more important factor for well-being in old age [18, 33]. However, the results of this study indicate that low tenacity, regardless of moderate flexibility, coincides with restricted life-space mobility. Thus, flexibility may function as an important resource for supporting sense of autonomy by helping adjustment to decreased outdoor mobility, while persistency may be more important for maintaining actual mobility and participation in community-life outside the home even when facing functional decline.

Earlier research has focused on studying FGA and TGP separately, even though they typically operate simultaneously in real-life situations [20]. Consequently, little knowledge exists on different coping profiles. Bailly et al. [21] identified the same three coping profiles, high TGP and high FGA, moderate TGP and low FGA, and low TGP and moderate FGA, as we did. We, however, found a fourth profile comprising those low in both TGP and FGA. This supports a recent finding indicating that those with low FGA are also likely to have low TGP [36]. Moreover, our participants were older than those studied by Bailly et al. [21] and interestingly, the participants in our cluster characterized by low tenacity and flexibility had the highest mean age (85.0 years). However, the suggested number of clusters was not perfectly stable, since one of the five analyses yielded only the first three profiles and not the fourth profile of low tenacity and low flexibility. This finding indicates the need for further research on coping profiles.

To our knowledge, this was the first study of coping strategies in relation to life-space mobility and perceived autonomy in participation outdoors. Thus, our findings contribute to the literature on the factors underlying out-of-home mobility in old age. We studied older people aged 79–93 years, since this is usually the time of life when people start to experience functional decline, which may limit their potential to achieve desired goals. We also studied TGP and FGA together as coping profiles, an approach that has rarely been taken hitherto. Another strength of this study was the population-based sample of community-dwelling older people without severe cognitive impairment and representative of all social strata. Data were collected with face-to-face interviews and missing values were few. Furthermore, we considered fundamental theory-based confounders in the analyses, and thus were able to examine whether physical or cognitive limitations, environmental barriers, and depressive symptoms affect the associations.

This study has also its limitations. The analyses were conducted in a cross-sectional dataset, and we were unable to investigate the temporal or causal relationships between the coping profiles and out-of-home mobility. Consequently, even though it is likely that active use of coping strategies underlies different aspects of outdoor mobility, we cannot be certain of that. Future studies should address the temporal associations and clarify which one is the predictor and which one is the outcome using a longitudinal study design. Furthermore, the coping profile of low TGP and low FGA was especially small, rendering it vulnerable to adjustments. Another limitation is that we assessed actual mobility behavior with self-report. However, the life-space mobility measure is well-established and validated [6, 10] and we took objectively measured confounders into account in the analyses, an approach that may enhance the validity of the findings. Finally, we did not use the short TGP and FGA scales in full, but removed the inversely phrased items as their validity has been found to be rather weak among older people [25].

## Conclusion

In old age, active use of both tenacious goal pursuit and flexible goal adjustment as coping strategies is beneficial for out-of-home mobility. Persistent goal pursuit, especially, seems to drive older people to participate in meaningful activities and community-life outside the home. In contrast, lack of tenacity and flexibility may restrict out-of-home mobility, a situation that is potentially explained by a higher prevalence of depressive symptoms. Future studies should investigate whether tenacious goal pursuit and flexible goal adjustment can predict changes in life-space mobility or in perceived autonomy in participation outdoors and whether they can be supported to promote out-of-home mobility among older people.

## Electronic supplementary material

Below is the link to the electronic supplementary material.

Supplementary material 1 (PDF 94 KB)
