# Supplementary material for: Association of tenacious goal pursuit and flexible goal adjustment with out-of-home mobility among community-dwelling older people
Source: Aging Clin Exp Res. 2018 Nov 17;31(9):1249–56. doi: 10.1007/s40520-018-1074-y (PMC6682663; doi:10.1007/s40520-018-1074-y)
Supplement: Supplementary file 1 — Supplementary material 1 (PDF 94 KB) [file 40520_2018_1074_MOESM1_ESM.pdf]

Association of Tenacious Goal Pursuit and Flexible Goal Adjustment with Out-of-Home Mobility Among Community-Dwelling Older People

Aging Clinical and Experimental Research

Sini Siltanen[1], MSc, Taina Rantanen[1], PhD., Erja Portegijs[1], PhD, Anu Tourunen[1], PhD, Taina Poranen-Clark[1], MSc, Johanna Eronen[1], PhD, Milla Saajanaho[1], PhD

[1] Gerontology Research Center and Faculty of Sport and Health Sciences, University of Jyväskylä, Jyväskylä, Finland

Corresponding author:
Sini Siltanen
E-mail: sini.k.t.siltanen@jyu.fi

Supplementary table. Items of the tenacious goal pursuit and flexible goal adjustment scales used in the current study, and the distribution of the responses in each item (n=186).

**Item**	**Response distribution %**				
	**0**	**1**	**2**	**3**	**4**
Tenacious goal pursuit					
1. Even when things seem hopeless, I keep on fighting to reach my goals	1	5	3	51	40
2. I stick to my goals and projects even in the face of great difficulties	1	13	6	44	36
3. The harder a goal is to achieve, the more appeal it has to me	6	19	16	33	25
4. I can be very stubborn/obstinate in pursuing my goals	2	13	9	39	36
Flexible goal adjustment					
1. If I do not get something I want, I take it with patience	0	9	6	37	48
2. I find it easy to see something positive even in a serious mishap	3	11	12	43	31
3. When everything seems to be going wrong, I can usually find a bright side to a situation	2	8	9	49	33
4. In general, I am not upset very long about an opportunity passed up	1	6	5	48	39

Note. The item scores range from strongly disagree (0) to strongly agree (4).
